# Expression of Notch1 Correlates with Breast Cancer Progression and Prognosis

**DOI:** 10.1371/journal.pone.0131689

**Published:** 2015-06-29

**Authors:** Xun Yuan, Mingsheng Zhang, Hua Wu, Hanxiao Xu, Na Han, Qian Chu, Shiying Yu, Yuan Chen, Kongming Wu

**Affiliations:** Department of Oncology, Tongji Hospital of Tongji Medical College, Huazhong University of Science and Technology, Wuhan, P.R. China; Stony Brook University, UNITED STATES

## Abstract

Various studies have evaluated the significance of Notch1 expression in breast cancer, but the results have ever been disputed. By using 21 studies involving 3867 patients, this meta-analysis revealed that the expression of Notch1 was significantly higher in breast cancer than in normal tissues (OR=7.21; 95%CI, 4.7-11.07) and that higher Notch1 expression was associated with transition from ductal carcinoma *in situ* (DCIS) to invasive cancer (OR=3.75; 95% CI, 1.8-7.78). Higher Notch1 activity was observed in the basal subtype of breast cancer (OR=2.53; 95% CI, 1.18-5.43). Moreover, patients with Notch1 overexpression exhibited significantly worse overall and recurrence-free survival. Our meta-analysis suggests that Notch inhibitors may be useful in blocking the early progression of DCIS and that the outcomes of clinical trials for Notch1-targeting therapeutics could be improved by the molecular stratification of breast cancer patients.

## Introduction

Breast cancer is the most common female cancer and represents 28.7% of all cancers diagnosed in woman [1]. With many advancements achieved in breast cancer biology and in diagnosis and treatment, the 5-year survival rates for local or regional breast cancer have increased to more than 85%. However, the majority of breast cancer patients with distant metastasis succumb to cancer progression within 5 years[1]. Therefore, the identification of biomarkers to screen high-risk patients and predict breast cancer outcomes in conjunction with traditional clinicopathological features is urgently needed.

A host of facts indicate that Notch pathway plays key roles in cell proliferation, differentiation and apoptosis. The role of Notch pathway in cancer was first revealed in T cell leukemia and breast cancer [2, 3]. Recently, our study indicated that higher expression of Notch signaling was associated with greater possibility of lymph node metastasis (LNM), higher TNM stages and poor survival of NSCLC patients [4]. During the development of breast cancer, increased expression of Notch1 was found and correlated with progression from hyperplasia to malignancy. The function that Notch signaling drives stemness and tumorigenicity was subsequently reported in breast cancer [5]. The significance of different isoforms of Notch in breast cancer is not clear, however, Notch1 is believed to be essential. Currently, one of the key goals is to evaluate the value of Notch signaling as a molecular marker in translational breast cancer research [6–14].

A number of studies have examined the correlation between Notch1 expression and clinical outcome in patients with breast cancer [15–18]. However, the prognostic value of Notch1 for breast cancer has yet to be confirmed. Some studies suggested that Notch1 overexpression foreboded a poor prognosis in breast cancer, but other researchers reported different results [19, 20]. Furthermore, Notch1 was usually utilized in combination with other biomarkers for the assessment of survival [21, 22]. Given the uncertainty of causality and inconsistencies among studies, a meta-analysis was performed to unearth the role of Notch1 in the clinicopathological features as well as prognosis of breast cancer. Notch activity in association with molecular subtypes of breast cancer was also analyzed.

## Materials and Methods

### Literature search

We searched the network databases PubMed, Embase and Cochrane library for studies published through October 12, 2014 using the search terms Notch (“Notch Receptors”,”Notch Proteins”) and breast cancer (“breast neoplasm”, “breast tumor”, “breast carcinoma”, “mammary cancer”). The references were also searched to discover extra relevant publications.

### Inclusion and exclusion criteria

This meta-analysis collected data from retrospective cohort studies aimed at evaluating the role of Notch1 expression in breast cancer. Literatures that met the following criteria were brought in: a) patients recruited in the study were pathologically diagnosed with breast cancer; b) Notch1 expression was measured in primary breast cancer tissue; c) the hazard ratio (HR) /odds ratio (OR) and corresponding 95% confidence interval (CI) were reported or could be statistically extracted from the study. The exclusion criteria were as follow: a) reviews, case reports, comments, letters and conference abstracts; b) ineligible samples or the required data were not available. When several articles were from the same patient population, the latest or most complete article was included.

### Data extraction

Data were abstracted using a standardized data collection form, with information recorded as follows: first author’s last name, publication year, country, histological type, number of included groups, detection methods, and cutoff values for Notch1. For articles without HRs, the statistical variables were calculated from published survival curves using the methods described by Tierney *et al*[23]. We also reviewed Oncomine dataset and identified 9 independent human breast cancer microarray datasets with Notch1 expression and survival data. Overall survival (OS), recurrence-free survival (RFS) and metastasis-free survival (MFS) were evaluated by Cox proportional HRs and 95% CIs using these numerical data.

For observational studies, the Newcastle-Ottawa Quality Assessment Scale (NOS) was employed to assess the quality of these studies. This scale is based on the identification of 8 sources of potential study bias and reflects patient selection, study comparability, and outcomes. The literature search, study selection, and data abstraction were performed independently by two reviewers, and disagreements between the reviewers were solved by discussion.

### Statistical analysis

Statistical analysis was conducted based on the requirements of the Meta-Analysis of Observational Studies [24]. The survival outcome of breast cancer patients with high Notch1 expression was evaluated by HRs and 95% CIs. The clinicopathological features included tumor size, histological type, TNM stage, pathological grade, the expression level of hormone estrogen receptor (ER), progesterone receptor (PR), and HER2. The OR and 95% CIs were used to evaluate the clinicopathological factors. Heterogeneity was appraised by means of the Cochran Q and I^2^test. The random-effect model was applied when heterogeneity was present (Cochran Q test p<0.1 or I^2^>50%). If heterogeneity was absent, a fixed-effect model was employed. Publication bias was assessed by Begg’s test and Egger’s test. All analyses were performed using the STATA software package (version 12.0) (Stata Corp LP, College Station, TX, USA).

## Results

### Search results

**Fig 1:**
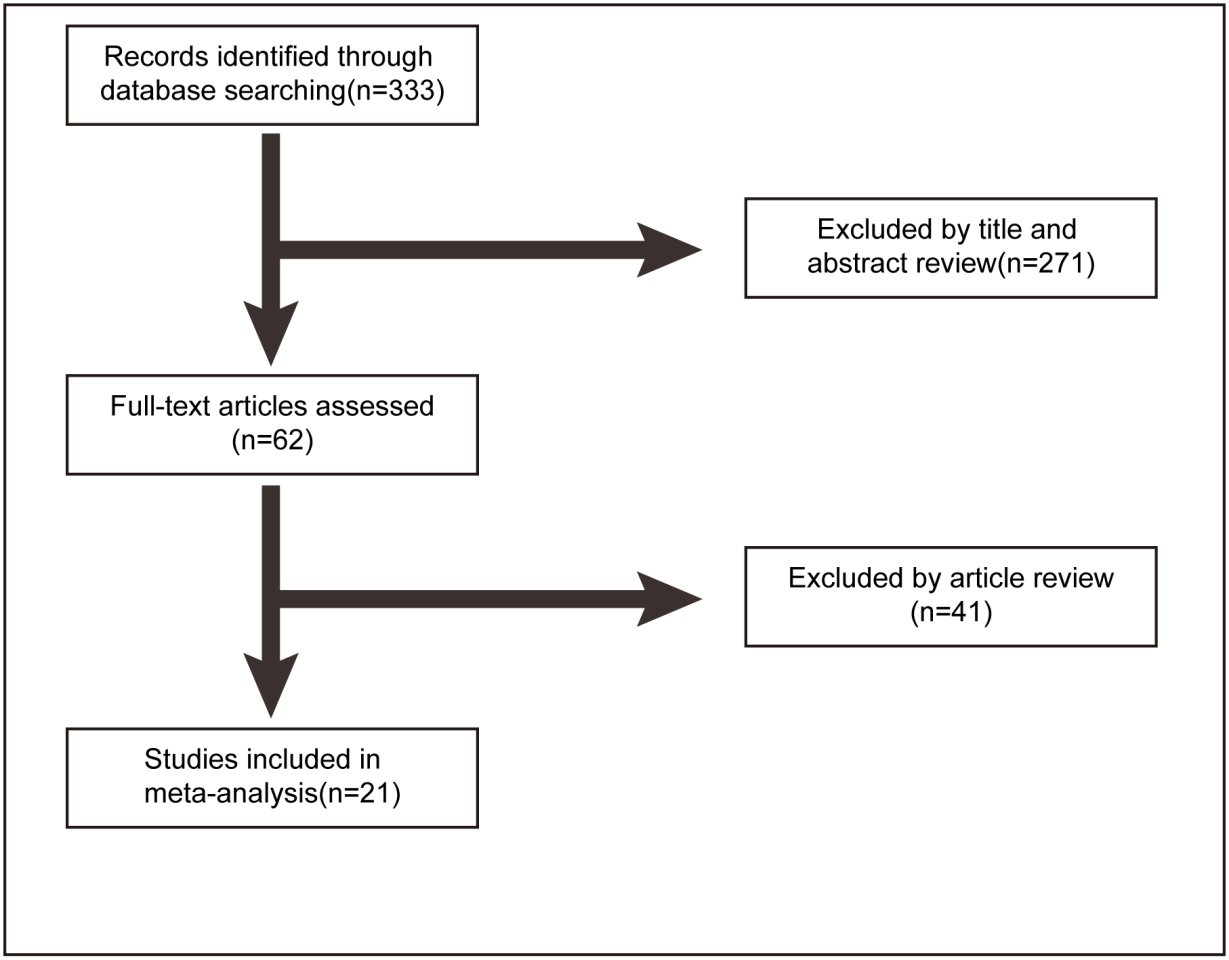
Flow diagram of article selection.

The flow diagram for the recognition of appropriate studies is presented in Fig 1. Three hundred thirty-three articles were identified by our search strategy. After the article titles, abstracts and full text were checked, 21 articles including 3867patientsmet the criteria for this analysis. The features of the 21 studies are listed in Table 1 and S1 Table. Clinical stage I and II disease were grouped as early staged disease; otherwise, III and IV were grouped as late staged disease. Histological grade I and II were pooled as low-grade disease, and III and IV were grouped as high–grade disease.

**Table 1:** Characteristics of the included studies in the meta-analysis.

First author	Year	Country or area	Duration(months)	Stage	Qualityscore	Detection	Cutoff values	Patients with Notch1 overexpression (Total NO)
Dong^26^	2007	China	NA	0-IV	8	RT-PCR	NA	61(62)
Desmedt^12^	2007	Canada	320	NA	9	Microarray	Average expression: 8.305	105(198)
Ercan^15^	2012	Netherlands	120	NA	9	IHC	Percentage of immunopositive cells >10%	334(449)
Farnie^8^	2007	UK	60	NA	9	IHC	Staining of H-score: 0–1 vs. 2–3	NA(50)
Hatzis^19^	2011	USA	90	I-IIIC	9	Microarray	Average expression:8.783	258(508)
Hua^27^	2009	China	NA	I-III	7	RT-PCR; IHC	RT-PCR (NA); Staining of H-score: 0–1 vs. 2–3	75(90)
Kao^17^	2011	Taiwan	170	NA	9	Microarray	Average expression:9.382	149(327)
Liu^28^	2009	USA	NA	NA	7	IHC	Staining of H-score: 0–1 vs. 2–3	8(24)
Loi^10^	2008	Canada	140	NA	9	Microarray	Average expression:0.018	38(77)
Ma^29^	2011	China	NA	NA	8	RT-PCR; IHC	RT-PCR (NA); Staining of H-score: 0–1 vs. 2–3	61(67)
Minn^14^	2005	USA	180	NA	9	Microarray	Average expression:452.7	19(82)
Mittal^22^	2009	India	NA	NA	7	IHC	Staining of H-score: 1–2 vs. 3–4	18(37)
Naderi^18^	2007	UK	180	NA	9	Microarray	NA	NA(135)
Pawitan^11^	2005	Sweden	120	NA	9	Microarray	Average expression:4.559	64(120)
Reedijk^16^	2005	Canada	300	NA	9	ISH; IHC	ISH(NA); Staining of H-score: 0–1 vs. 2–3	NA(170)
Rizzo^6^	2008	USA	NA	NA	9	IHC	Staining of H-score: 0–1 vs. 2–3	54(73)
Schmidt^13^	2008	Germany	240	NA	9	Microarray	Average expression:245.166	86(200)
Wang^20^	2005	USA	180	NA	9	Microarray	Average expression:400.848	112(286)
Yao^25^	2011	USA	NA	NA	9	IHC	Staining of H-score: 0–1 vs. 2–3	30(48)
Zardawi^9^	2010	Australia	180	NA	8	IHC	Staining of H-score: 0–1 vs. 2–3	NA(724)
Zhang^7^	2011	China	NA	NA	7	IHC	Staining of H-score: 0–1 vs. 2–3	106(140)

Abbreviation: NA, not available; NO, number; IHC, immunohistochemistry; RT-PCR, real-time reverse transcriptionpolymerase chain reaction; ISH, in situ hybridization.

### Notch1 expression positively correlates with breast cancer progression

**Fig 2:**
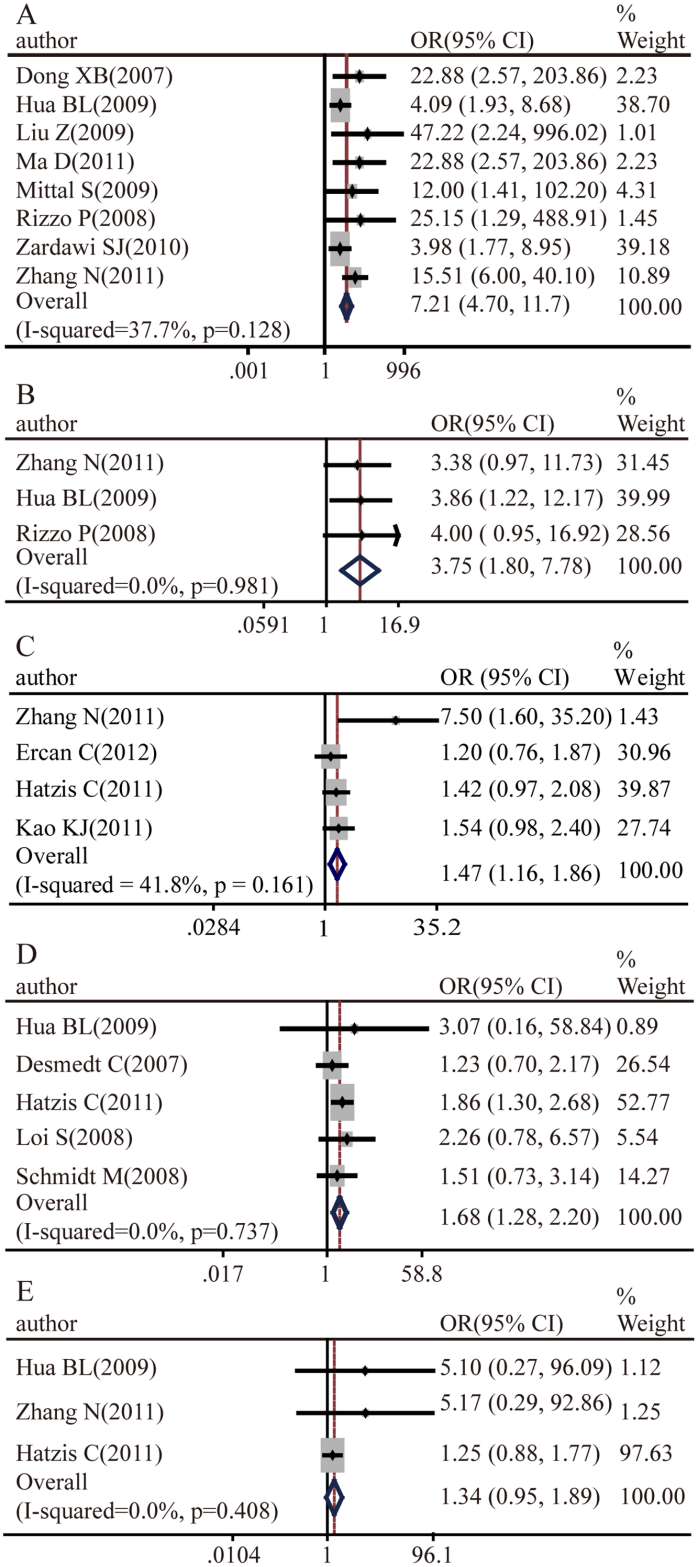
Correlation between Notch1 expression and breast cancer development and progression as evaluated by the odds ratio (OR). **A.** Association between Notch1 and breast cancer risks compared with normal breast tissues. **B.** Association between Notch1 and invasive ductal cancer risk compared with DCIS. **C.** Association between Notch1 and lymphatic metastasis. **D.** Association between Notch1 and tumor grade. **E.** Association between Notch1 and clinical stages (tumor size).

Totally, 18 studies have assessed the association between Notch1 expression and tumor clinicopathological features. Our meta-analysis demonstrated that Notch1 expression in breast cancer tissues was significantly higher than that in normal breast tissues (pooled OR = 7.21, 95%CI: 4.7–11.07, Cochran Q’s test p = 0.128 and I^2^ = 37.7%) (Fig 2A). Notch1 was positively related to progression, exhibiting elevated expression in invasive ductal cancer (IDC) relative to carcinoma *in situ*(pooled OR = 3.75, 95%CI: 1.8–7.78, p = 0.981 and I^2^ = 0.0%) (Fig 2B). Moreover, an increased likelihood of LNM and higher histological grade were correlated with high Notch1 expression in breast cancer (pooled OR = 1.65, 95%CI: 1.09–2.52, p = 0.064 and I^2^ = 55%; pooled OR = 1.68, 95%CI: 1.28–2.2, p = 0.737 and I^2^ = 0.0%) (Fig 2C and 2D). However, no statistically significant correlation between Notch1 expression and tumor stage (I-II, III-IV) was found (pooled OR = 1.34, 95%CI: 0.95–1.89, p = 0.408 and I^2^ = 0.0%) (Fig 2E).

### Notch1 was enriched in the basal subtype of breast cancer

**Fig 3:**
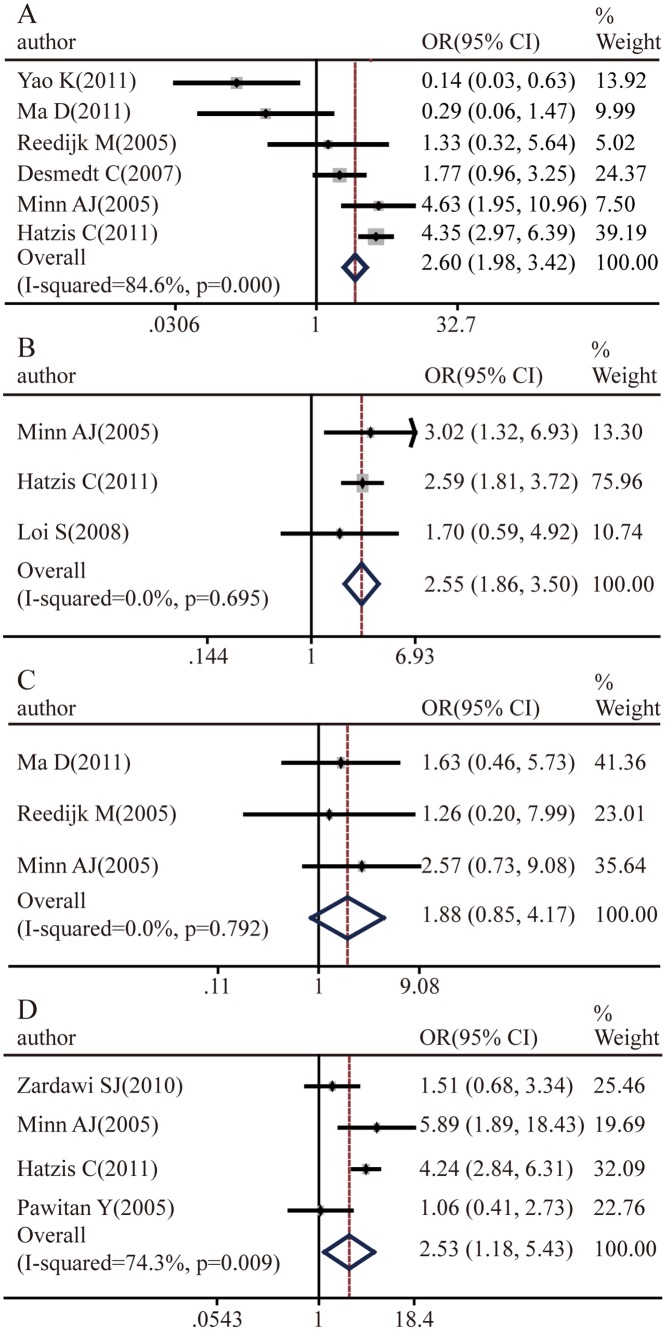
Notch1 expression levels with respect to molecular subtypes. **A.** Association between Notch1 and ER-negative breast cancer risk compared with ER-positive breast cancer and normal breast tissues. **B.** Association between Notch1 and PR-negative breast cancer risk compared with PR-positive breast cancer and normal breast tissues. **C.** Association between Notch1 and the basal subtype of breast cancer relative to luminal breast cancer. **D.** Association between Notch1 and Her2-positive breast cancer.

The association of Notch1 expression with breast cancer molecular subtype was also analyzed. Notch1 was inversely correlated with ER expression (pooled OR = 2.60, 95%CI: 1.98–3.42, p = 0.000 and I^2^ = 84.6%) (Fig 3A) and PR expression (pooled OR = 2.55, 95%CI: 1.86–3.5, p = 0.695 and I^2^ = 0.0%) (Fig 3B). There was no significant relationship between Notch1 and Her2 status (pooled OR = 1.88, 95%CI: 0.85–4.17, p = 0.792 and I^2^ = 0.0%) (Fig 3C). However, the expression of Notch 1 was enriched in the basal subtype of breast cancer (pooled OR = 2.53, 95%CI: 1.18–5.43, p = 0.009 and I^2^ = 74.3%) (Fig 3D).

### Notch1 expression effects on breast cancer survival

**Fig 4:**
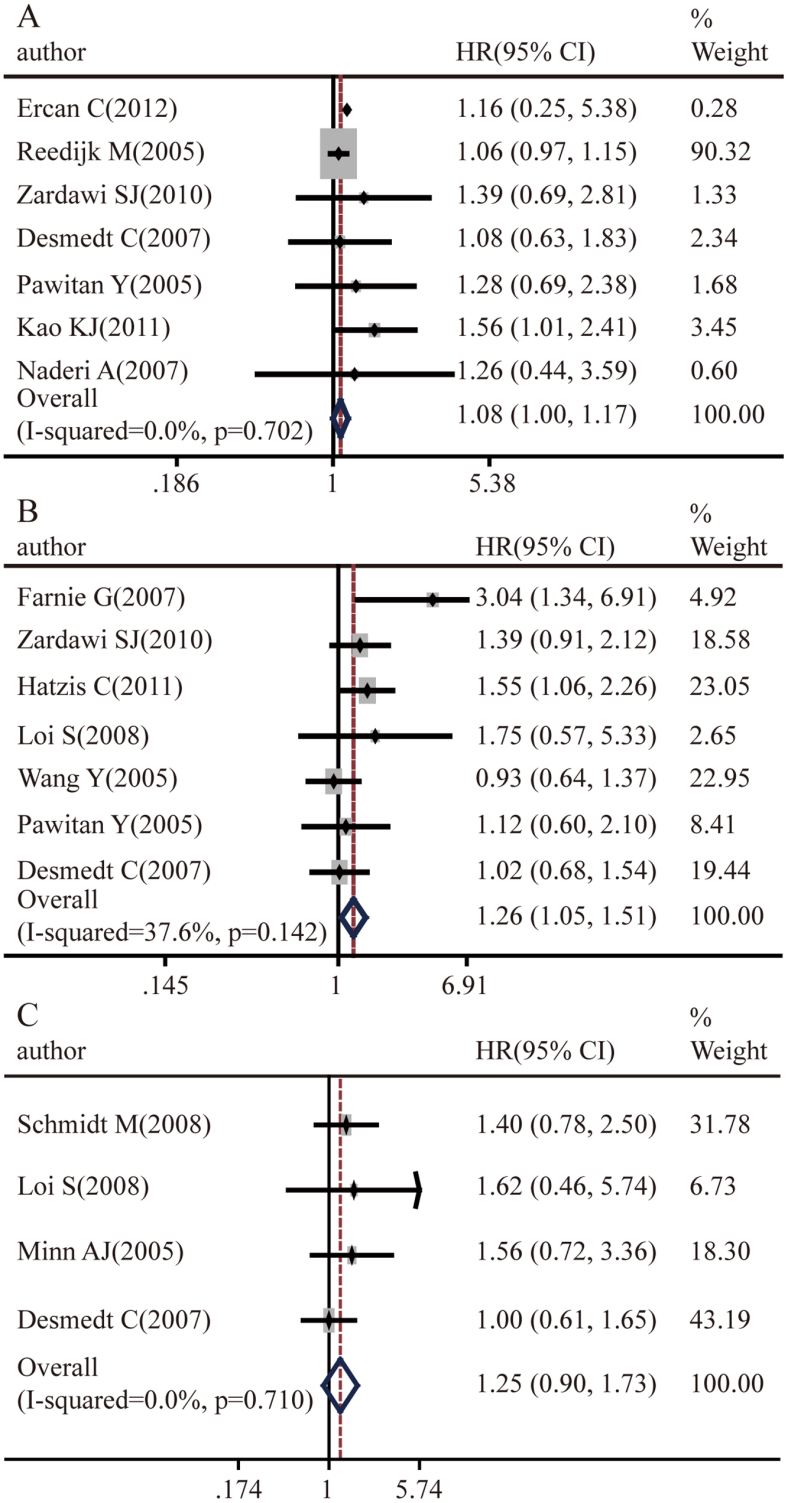
Elevated Notch1 protein abundance correlates with poor prognosis. **A.** Association between Notch1 and breast cancer OS. **B.** Association between Notch1 and breast cancer RFS. **C.** Association between Notch1 and MFS.

**Fig 5:**
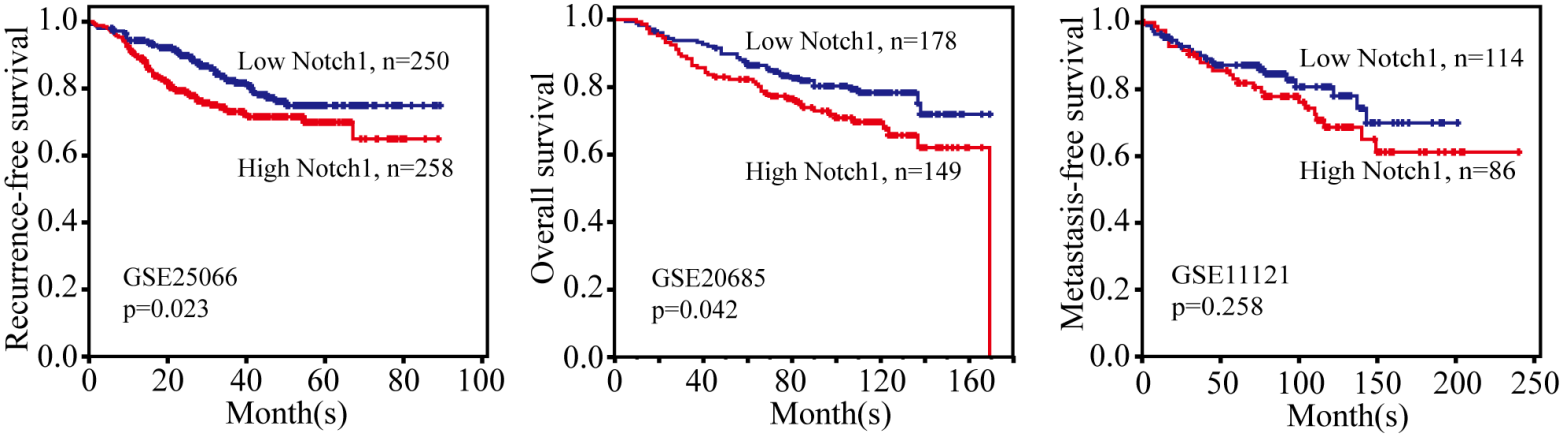
Correlation of Notch1 mRNA expression with breast cancer survival. **A.** RFS rate with respect to Notch1 expression was analyzed in 508 patients (GSE25066) with breast cancer. **B.** OS rate with respect to Notch1 expression analyzed in 327 patients (GSE20685) with breast cancer. **C.** MFS rate with respect to Notch1 expression was analyzed in 200 patients (GSE11121) with breast cancer.

Ultimately, we evaluated the association between Notch1 expression level and survival outcome of breast cancer patients (Fig 4 and S1 Fig). The results indicated that Notch1 overexpression was statistically associated with the OS rate (pooled HR = 1.08, 95%CI: 1.00–1.17, p = 0.702 and I^2^ = 0.0%)(Fig 4A) and RFS rate (pooled HR = 1.26, 95%CI: 1.05–1.51, p = 0.142 and I^2^ = 37.6%) (Fig 4B). However, there was no significant relationship between Notch1 expression and MFS (Fig 4C). Subcategory analyses according to the molecular classification of breast cancer were also performed, but we did not find significant difference in survival outcome between basal-like breast cancer and luminal breast cancer for the limited sample size. To investigate whether Notch1 mRNA abundance correlated with breast cancer survival, we analyzed published gene expression databases of breast cancer from Oncomine with survival information. Kaplan–Meier survival analysis of GSE25066, which contained 508 breast cancer patients, demonstrated that RFS rate was lower in patients with high Notch1 expression(p = 0.024) (Fig 5A). Similarly, the OS rate was also inversely associated with Notch1 expression(p = 0.044) (Fig 5B). However, there was no significant effect of Notch1 expression on MFS (p = 0.261) (Fig 5C). Together, the results from the Notch1 mRNA profile are consistent with those of protein abundance, both indicating that higher Notch1 activity predicts a worse prognosis.

### Publication bias

Publication bias statistics were obtained using Begg’s test and Egger’s test, which did not indicate any significant publication bias: breast cancer: Begg’s test p = 1.000, Egger’s test p = 0.675; basal breast cancer: Begg’s test p = 1, Egger’s test p = 0.738; IDC: Begg’s test p = 0.296, Egger’s test p = 0.129; tumor size: Begg’s test p = 0.806, Egger’s test p = 0.8; histological grade: Begg’s test p = 1, Egger’s test p = 0.133; LNM: Begg’s test p = 0.368, Egger’s test p = 0.615; expression of ER: Begg’s test p = 1, Egger’s test p = 0.878; expression of PR: Begg’s test p = 1, Egger’s test p = 0.379; expression of Her2: Begg’s test p = 0.734, Egger’s test p = 0.513; OS: Begg’s test p = 1, Egger’s test p = 0.096; RFS: Begg’s test p = 0.368, Egger’s test p = 0.245; MFS: Begg’s test p = 0.734, Egger’s test p = 0.291; OS of luminal breast cancer: Begg’s test p = 0.308, Egger’s test p = 0.238; RFS of luminal breast cancer: Begg’s test p = 1, Egger’s test p = 0.843; and MFS of luminal breast cancer: Begg’s test p = 1, Egger’s test p = 0.682;

## Discussion

It is generally believed that Notch1 can be used as a progressive biomarker for breast cancer [25–29]. Precisely measuring the prognostic value of Notch1 may help to guide individual therapies for breast cancer patients. This meta-analysis suggested a predictive value of Notch1 expression in breast cancer patients. Notch1 expression is significantly associated with invasive ductal carcinoma, lymphatic metastasis and histological grade. In addition, our meta-analysis suggested that Notch1 overexpression could be a prognostic marker for OS and RFS. However, we failed to identify an association between Notch1 expression and MFS. Multiple signaling molecules, including epithelial growth factor receptor (EGFR), vascular endothelial growth factor (VEGFR) and transforming growth factor β (TGF-β), participate in the metastatic process[30]. Breast cancer patients with coordinate activation of Notch and Ras/MAPK signaling demonstrated early relapse and poor OS, and combined inhibition of both pathways revealed synergistic anti-tumor effects [31]. Considering the cross-talk between Notch with multiple pathways, we surmise that the influence of Notch1 on MFS was reduced in patients with advanced stage breast cancer, most likely due to the redundancy and complexity of diverse signaling pathways [8, 15, 22].

Notch1signaling regulates multiple functions, including proliferation/differentiation, motility, cell-cell connections and cell polarity [32, 33]. Thus, it is predictable that Notch1 activity could affect invasion, lymphatic metastasis and histological grade [34, 35]. We found that Notch1 expression increased approximately 7-fold in breast tumor tissues (OR = 7.21; 95% CI, 4.7–11.7), indicating that Notch signaling is essential in breast tumorigenesis. Our analysis also revealed that Notch1 was a critical molecule for the switch from DCIS to invasive cancer (OR = 3.75; 95% CI, 1.8–7.78). This observation is supported by recent laboratory finding that Notch could regulate EMT and invasion [36], strongly suggesting that early prevention of DCIS with theNotch1 inhibitor might reduce progression. Breast cancer can be divided into 3 major subtypes that exhibit distinctive expression patterns of specific molecules, clinical outcomes, and responses to adjuvant chemotherapy [17, 37]. Identification of the relationship between Notch signaling and other pathways in breast cancer is vital to decide which patients would benefit from Notch inhibitors at most. The cross-talk between Notch1 and ER in breast cancer leads to the decreased expression of Notch1 in ER-positive cells [6]. In agreement, our meta-analysis revealed that Notch1 abundance was negatively associated with the expression of ER/PR. However, patients with activation of Notch pathway demonstrated acquired resistance to tamixifen treatment [6, 38], indicating that combinations of anti-estrogens and Notch inhibitors may be effective in ER(+) breast cancers [6]. In contrast, higher Notch1 activity was observed in basal type breast cancer, suggesting that theNotch1 inhibitor could be more likely to benefit patients with basal-type breast cancer. In support of this finding, an experimental study demonstrated that the Notch inhibitor induced more apoptosis in basal cells than in luminal cells [39]. Indeed, triple-negative breast cancer exhibits distinctive patterns of Notch expression, and EGFR and Ki-67 are positively associated with Notch1 [40]. Recent clinical study in triple negative breast cancer has identified PEST domain mutation in Notch1, leading to the activation of Notch pathway. Moreover, high sensitivity to gamma secretase inhibitor (GSI) PF-03084014 was observed in xenograft model from patient-derived cancer with Notch1 mutation [41].

Increasing evidence suggests that cancer stem cells (CSCs) are responsible for cancer therapy resistance, metastasis and relapse. Ectopic expression of Notch1 in luminal breast cancer cell MCF-7 and immortalized breast luminal epithelial cell MCF10A induced EMT and acquired CSC phenotypes [42]. In the basal-likeMDA-MB-231 breast cancer cell model, the subpopulation of CD44 (+) CD24 (low+) cells exhibits stem cell properties *in vitro* and *in vivo*, metastatic gene signatures and greater invasion and metastatic potential. Interestingly, this subpopulation of cells also expresses activated Notch1 intracellular domain (N1-ICD) and Notch1 target genes. Moreover, GSI decreased sphere formation and xenograft growth from CD44 (+) CD24 (low+) cells [43]. The identification of biomarkers for micro-metastases, disseminated tumors, and residual disease is critical for the early detection and treatment of these diseases prior to their full development into metastases and recurrent tumors. In the MMTV-PyMT breast cancer model, disseminated tumor cells revealed enrichment of the Notch pathway. Thus, these results suggest that Notch pathway may be involved in metastasis and tumor relapse after therapy [44]. Treatment with the Notch1 antibody resulted in decreased rates of tumorigenesis and tumor recurrence, demonstrating the potent antitumor efficacy of a Notch1 antibody and remarkable activity against CSCs. These findings suggest that blocking Notch1 activity may represent a novel therapy to improve the therapeutic effects of conventional therapies, thus delaying tumor recurrence and improving cancer patient survival [45].

Currently, there is no effective target therapy for the basal type breast cancer. The identification of novel therapeutic targets for these patients with poor prognoses will be critical to further improve breast cancer treatment. *In vitro*, GSIPF-03084014 inhibited tumor cell migration and endothelial cell tube formation as well as mammosphere formation. On the other hand, treatment with PF-03084014 induces apoptosis, reduces tumor cell self-renewal ability, impairs tumor vasculature, and decreases metastasis activity. Molecular analysis also revealed that Notch pathway target genes, HEY2, HES4, and HES3, strongly correlated with the effect of GSI [46].

Heterogeneity tests are an essential part of meta-analysis. In this study, evidence of minor heterogeneities was observed with respect to OS, RFS and MFS; however, there was substantial heterogeneity with respect to tumor size and molecular subtype. This unbalanced result could partly arise from the limited sample size, indicating that multicenter prospective studies are needed. Another significant heterogeneity was likely due to the variations in assessing Notch1 expression. The cutoff value was estimated in 9studies using the average Notch1 level measured by gene microarray, whereas the cutoff values in the remaining 11 articles were determined by IHC. Some studies defined Notch1 expression based upon the percentage of immunopositive cells or staining intensity, whereas other studies used a scoring system that combined those 2 factors, creating diversity in dectecting cutoff values for Notch1 expression. Lastly, publication bias is worth considering in meta-analyses. In this study, only articles reporting clinical stage exhibited publication bias, whereas analyses of OS, RFS, MFS and other clinicopathological parameters did not display large variations. However, there are still some defects in this meta-analysis. First of all, the relevant papers were limited. Secondly, researchers used different method and cutoff values to assess Notch1 expression. Lastly, we cannot ignore the publication bias. Some articles are still unavailable.

This meta-analysis suggests that Notch1 is an important indicator of progression and is associated with prognosis, especially in the basal type of breast cancers. This result is sustained by a recent study that silencing Notch1 can inhibit the growth of tumor xenografts in animal models of breast cancer [47]. Our conclusion was also supported by a recent study using Sirt1-low/N1C-high as a prognosis factors, double parameters may provide more precision information [48]. Apart from being a prognostic biomarker, Notch1 may be used as a molecular target [6, 25, 32, 33]. Currently, several classes of Notch inhibitors have been developed, and promising results have been observed in some patients in clinical trials. However, no satisfactory molecules are universally accepted as biomarkers to guide patient selection [33]. Our analyses combined with laboratory results strongly suggest that basal-type breast cancers are most likely benefit from treatment with Notch inhibitors. Importantly, next-generation sequencing identified high frequency rearrangements of Notch1 and Notch2 in triple negative breast cancer (6 of 66 tumors), which leading to constitute receptor activation; interestingly, cell lines with this genetic changes were sensitive to GSI treatment by in vitro and in vivo assays [49]. It is expected that patient selection guided by molecular or genetic characteristics would greatly improve outcome in future GSIs study.

## Supporting Information

S1 Fig Forest plot of subgroup analysis by test method for Notch1 expression. (PDF)

S1 Table Characteristics of the included human breast cancer microarray datasets in the meta-analysis. (DOCX)
