# Supplementary material for: Expression of Notch1 Correlates with Breast Cancer Progression and Prognosis
Source: PLoS One. 2015 Jun 29;10(6):e0131689. doi: 10.1371/journal.pone.0131689 (PMC4488260; doi:10.1371/journal.pone.0131689)
Supplement: S1 Fig — (PDF) [file pone.0131689.s001.pdf]

Supplementary Figure S1

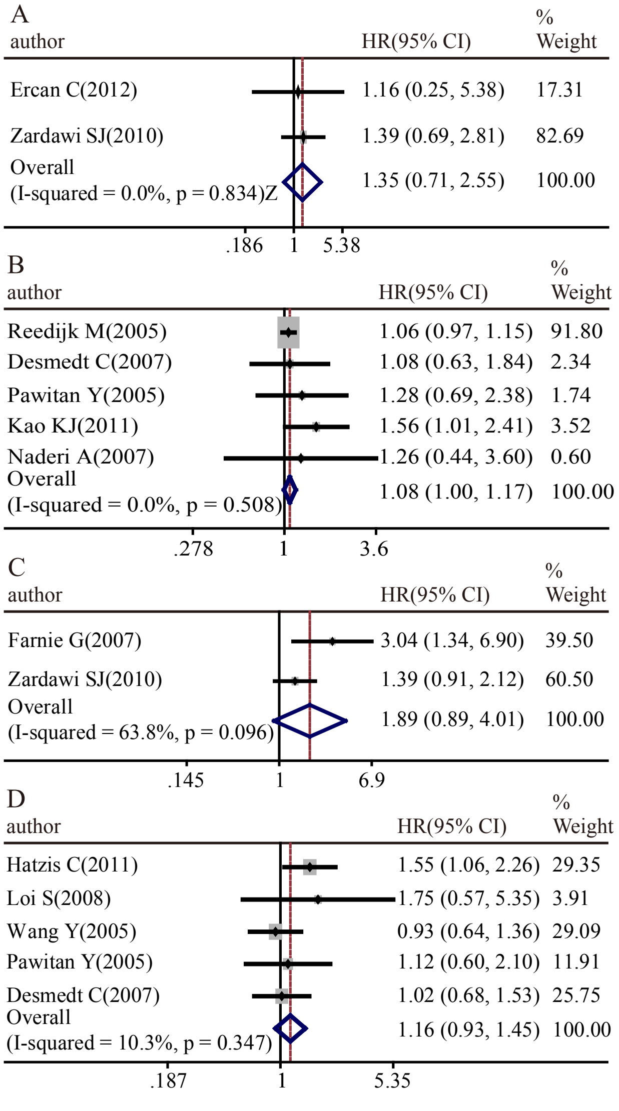

Supplementary Figure S1. Forest plot of subgroup analysis by test method for Notch1 expression.
A. Association between Notch1 expression and breast cancer OS measured by IHC.
B. Association between Notch1 expression and breast cancer OS measured by gene expression array and qPCR.
C. Association between Notch1 expression and breast cancer RFS measured by IHC.
D. Association between Notch1 expression and breast cancer RFS measured by gene expression array and qPCR.
