# Supplementary material for: Expression of Notch1 Correlates with Breast Cancer Progression and Prognosis
Source: PLoS One. 2015 Jun 29;10(6):e0131689. doi: 10.1371/journal.pone.0131689 (PMC4488260; doi:10.1371/journal.pone.0131689)
Supplement: S1 Table — (DOCX) [file pone.0131689.s002.docx]

**Supplementary Table 1.** Characteristics of the included human breast cancer microarray datasets in the meta-analysis.

| Study | Patients No | Median survival time (%95CI) | P value | GEO Accession |
| --- | --- | --- | --- | --- |
| Desmedt^12^ | 198 | RFS:193.9(159.9,227.8)  MFS:239.5(203.4,275.6) | 0.806  0.918 | GSE7390 |
| Hatzis^19^ | 508 | N.R. | N.R. | GSE25066 |
| Kao^17^ | 327 | OS:169.2(125.8,212.6) | 0.042 | GSE20685 |
| Loi^10^ | 77 | N.R. | N.R. | GSE9195 |
| Minn^14^ | 121 | N.R. | N.R. | GSE2603 |
| Naderi ^18^ | 135 | N.R. | N.R. | E-UCON-1 |
| Pawitan^11^ | 159 | N.R. | N.R. | GSE1456 |
| Schmidt^13^ | 200 | N.R. | N.R. | GSE11121 |
| Wang^20^ | 286 | N.R. | N.R. | GSE2034 |

Abbreviation: N.R., not reached median survival time.
